# Cross-Species Studies Reveal That Dysregulated Mitochondrial Gene Expression and Electron Transport Complex I Activity Are Crucial for Sarcopenia

**DOI:** 10.3390/ijms251910302

**Published:** 2024-09-25

**Authors:** Ji-Yoon Lee, Su-Kyung Shin, Ji-Won Han, Eun-Young Kwon, Heekyong R. Bae

**Affiliations:** 1 Department of Food Science and Nutrition, Kyungpook National University, 80, Daehak-ro, Buk-Ku, Daegu 41566, Republic of Koreassk1210@knu.ac.kr (S.-K.S.);; 2 Center for Food and Nutritional Genomics Research, Kyungpook National University, 80, Daehak-ro, Buk-Ku, Daegu 41566, Republic of Korea; 3 Center for Beautiful Aging, Kyungpook National University, 80, Daehak-ro, Buk-Ku, Daegu 41566, Republic of Korea

**Keywords:** aging, electron transport complex I, macrophage, mitochondria, muscle, skeletal, sarcopenia

## Abstract

The significance of complex I of the electron transport chain (ETC) in the aging process is widely acknowledged; however, its specific impact on the development of sarcopenia in muscle remains poorly understood. This study elucidated the correlation between complex I inhibition and sarcopenia by conducting a comparative analysis of skeletal muscle gene expression in sarcopenia phenotypes from rats, mice, and humans. Our findings reveal a common mechanistic link across species, particularly highlighting the correlation between the suppression of complex I of ETC activity and dysregulated mitochondrial transcription and translation in sarcopenia phenotypes. Additionally, we observed macrophage dysfunction alongside abnormal metabolic processes within skeletal muscle tissues across all species, implicating their pathogenic role in the onset of sarcopenia. These discoveries underscore the importance of understanding the shared mechanisms associated with complex I of ETC in sarcopenia development. The identified correlations provide valuable insights into potential targets for therapeutic interventions aimed at mitigating the impact of sarcopenia, a condition with substantial implications for aging populations.

## 1. Introduction

Sarcopenia, which denotes the age-associated loss of muscle mass, strength, and function, has emerged as a significant biomedical challenge, particularly given its implications for physical well-being and overall health in the elderly population [1]. The underlying mechanisms of sarcopenia encompass a complex interplay of factors, including dysregulation in protein synthesis and degradation, perturbation in mitochondrial function, insulin resistance, and immune aging, also known as immunosenescence, referring to the gradual deterioration of the immune system with age [2,3,4,5].

Among these factors, mitochondrial dysfunction plays a significant role in the aging process and is closely linked to the development of sarcopenia [6]. With advancing age, there is a gradual decline in mitochondrial function, leading to reduced ATP production, which can contribute to the overall decline in cellular function associated with aging. This energy deficit was also observed in sarcopenia, emphasizing the intricate connection between mitochondrial dysfunction, aging, and the pathogenesis of muscle wasting [7]. Aging-induced damage to muscle mtDNA leads to mitochondrial dysfunction, resulting in reduced ATP production. This ultimately correlates closely with aerobic capacity and glucose intolerance, impacting the muscle function changes observed in the elderly. Mitochondrial dysfunction, which is characterized by increased production of reactive oxygen species (ROS) during ETC reactions, particularly in complex I and III, further exacerbates the aging process and contributes to sarcopenia [8,9].

Mitochondrial respiratory complex I receives electrons from NADH during oxidative phosphorylation (OXPHOS) and generates energy through the ETC [9]. While inhibition of complex I by aging has been reported in numerous studies [10,11], the mechanistic understanding of this phenomenon has yet to be well established [12,13]. Increased ROS resulting from the inhibition of complex I of ETC can induce mutations in mitochondrial DNA within cells in neurodegeneration [14]. This, in conjunction with the functional decline of mitochondria, may impact mitochondrial biogenesis and mitophagy, the selective removal of damaged mitochondria. Nevertheless, these aspects are surrounded by controversy and require further clarification [15]. More research is needed to understand the impact of inhibition of complex I of ETC, particularly on sarcopenia.

Therefore, this study aimed to elucidate how mitochondrial respiratory complex I activity, in particular, contributes to the pathogenesis of sarcopenia in the correlation between inflammation, mitochondrial dysfunction, and sarcopenia. This will be achieved through a comparative analysis of skeletal muscle gene expression derived from elderly rats, mice, and humans. The ages of the rat, mouse, and human were 24 months, 28 months, and 73 years, respectively. The gene analyzed in the rat and mouse was from the gastrocnemius, while in the human, it was from the left vastus lateralis. By examining the associated mechanisms, we seek to unravel the pathogenesis of sarcopenia and gain insights into the processes involved.

## 2. Results

### 2.1. Common Pathways across Species Based on Hallmark Gene Set Analysis Using DEGs or Ranked Gene Lists 

**Figure 1:**
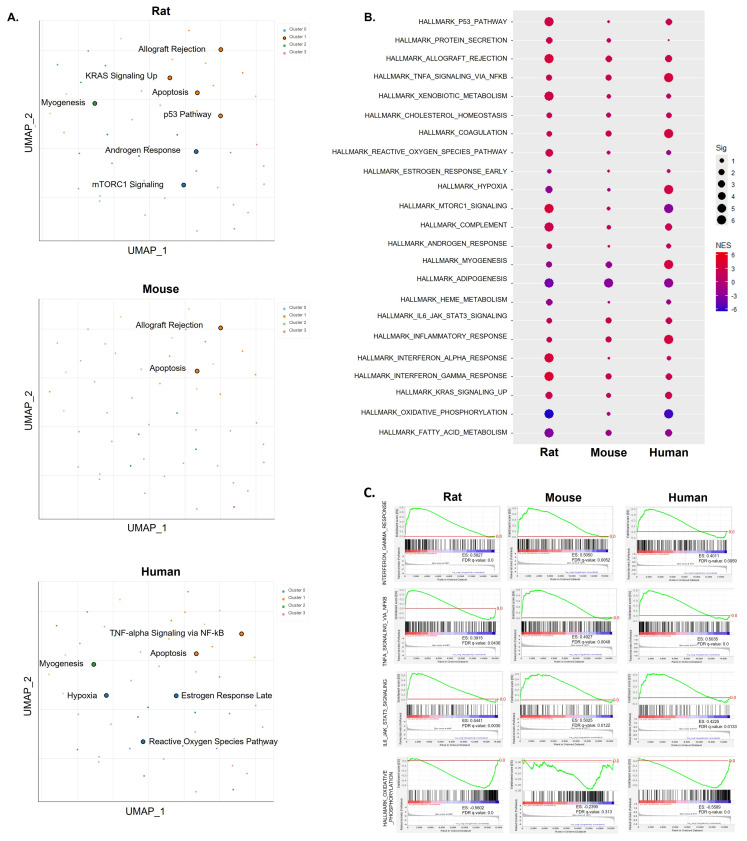
Common pathways across species based on hallmark gene set analysis in skeletal muscle. (**A**) UMAP displaying differentially expressed genes (DEGs) in skeletal muscle from rats, mice, and humans. Significantly enriched gene sets are highlighted by larger, black-outlined points. (**B**) Dot plots showing pre-ranked GSEA results in skeletal muscle from across species. The dot size indicates significance (−log10(FDR *q*-value)); the dot color represents the NES. (**C**) Representative plots showing enrichment in inflammation and lipid metabolism generated from GSEA across species. The baseline (0.0) of the enrichment score is indicated by a red color line.

To explore the significant pathways based on DEGs, we conducted hallmark gene set analysis using Enrichr. We characterized overlapped pathways in the skeletal muscle of rats, mice, and humans with sarcopenia (Figure 1A). However, strongly overlapping pathways were limited, with apoptosis being the primary exception across species. Despite this, the pathways detectable in each species exhibited an overall correlation with the pathogenic processes of sarcopenia. Myogenesis, mTOR signaling, apoptosis, the p53 pathway, TNF-α signaling via NF-κB, hypoxia, and the ROS pathway directly or indirectly influence sarcopenia. Subsequently, we employed whole gene expression profiles to identify common pathways across species through pre-ranked GSEA (Figure 1B). Notably, among hallmark gene sets, p53 pathway, allograft rejection, TNF-α signaling via NF-κB, IL-6 JAK STAT3 signaling, inflammatory response, IFN-γ response, IFN-α response, and KRAS signaling up were commonly elevated across all three species. Among these, inflammatory responses, such as IFN-γ, IFN-α, TNF-α, and IL-6 responses, were consistently positively enriched, as illustrated in Figure 1C. Another significant change was observed in lipid metabolic pathways, encompassing oxidative phosphorylation and fatty acid metabolism in Figure 1B. Overall, these findings suggest a correlation with the pathogenic processes of sarcopenia and provide evidence of shared molecular mechanisms across different species.

### 2.2. The Abnormal Metabolic Process Was Linked to Mitochondrial Respiratory Complexes across Species

**Figure 2:**
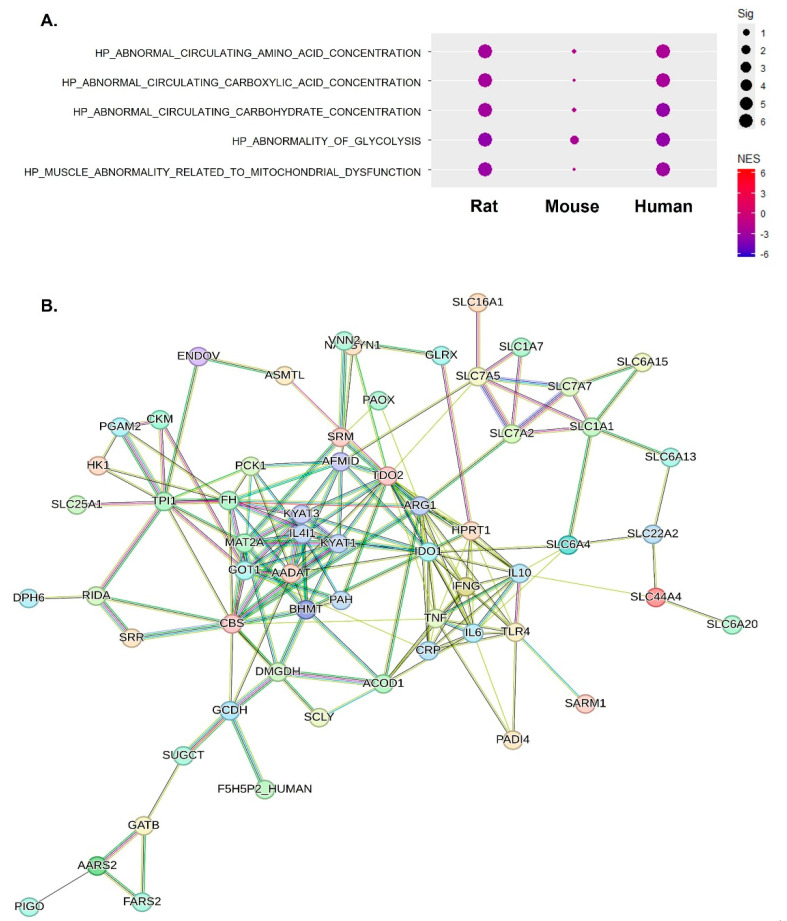
Abnormal metabolic processes associated with inflammatory responses in sarcopenia across species. (**A**) Dot plots demonstrating the enrichment of gene sets related to abnormal metabolic processes. The dot size corresponds to the significance level (−log10(FDR *q*-value)); the dot color indicates the NES. (**B**) Network analysis of significant metabolites in plasma from aged mice with the addition of IFN-γ, TNF-α, IL-6, IL-10, and TLR4 responses that are detectable across species.

**Figure 3:**
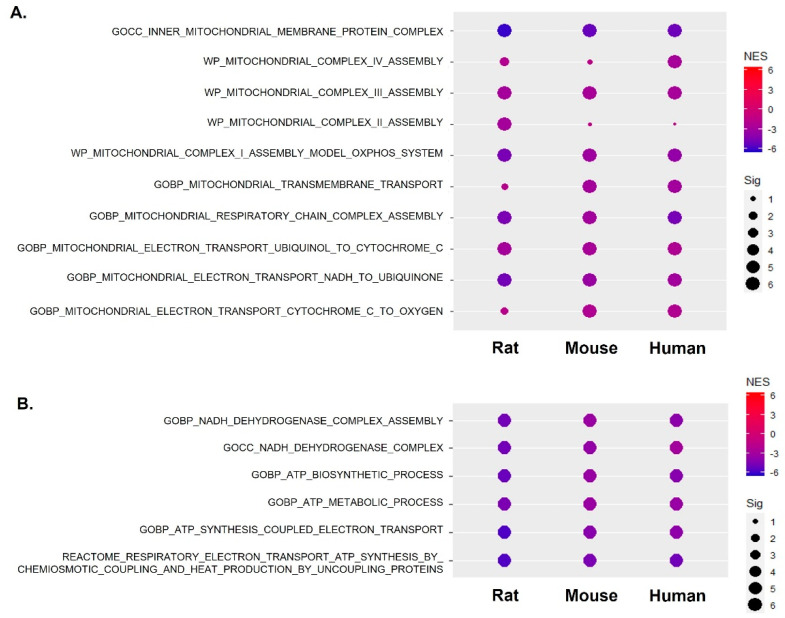
Mitochondrial dysfunction with the suppression of electron respiratory complex I in sarcopenia across species. (**A**) Dot plots demonstrating the enrichment of gene sets related to mitochondrial respiratory complex across species. (**B**) Dot plots demonstrating the enrichment of gene sets related to mitochondrial NADH and ATP across species.

We conducted a more in-depth characterization of alterations in various metabolic processes using MSigDB. In Figure 2A, we present the changes in circulating amino acid, carboxylic acid, carbohydrate concentration, and glycolysis and changes related to mitochondrial dysfunction. Both rat and human gene sets displayed negative enrichment with significant enrichment scores (ES) and FDR *q*-values. Although the mouse data did not achieve significant FDR *q*-values, a closer examination of the gene set bar placements—where each bar represents a gene—revealed a similar pattern of negative enrichment, aligning with the results seen in rats and humans. Overall, these data indicate that the genes involved in each metabolic process were downregulated across species. The GSEA graph in Supplementary Figure S1A provides a detailed representation of circulating carbohydrate concentration and glycolysis, which was expected to be strongly correlated with mitochondrial dysfunction in Figure 3. Accordingly, as a result of network analysis using the genes included in these pathways, FOXRED1, NDUFS1, NDUFS8, COQ9, MPC1, NDUFS3, and NDUFS7 in the rat constituted the center of the network. FOXRED1, TMEM126B, NDUFB11, and NDUFS4 in the mouse and FOXRED1, TIMMDC1, NDUFAF4, NDUFV1, NDUFS6, and NUBPL in the human constituted the core of the network (Supplementary Figure S1B). The list of genes associated with changes in metabolic processes was strongly correlated with genes involved in mitochondrial function, particularly mitochondrial respiratory complex I activity. This correlation was consistently observed in the aged skeletal muscle from rats, mice, and humans. Furthermore, we leveraged differentially expressed metabolomics data obtained from the plasma of elderly men and the skeletal muscle of aged mice. Through this analysis, we elucidated a central connection between the inflammatory responses mediated by IFN-γ, TNF-α, IL-6, IL-10, and TLR4, and these identified metabolites (Figure 2B). In summary, these findings suggest that there is a correlation between metabolic alterations and changes in mitochondrial complex I in aged skeletal muscle. The suppression of complex I may be interconnected, with systemic metabolic shifts induced by chronic inflammatory responses.

### 2.3. Mitochondrial Complex I Activity along with Mitochondrial Gene Expression Was Significantly Suppressed across Species

To elucidate the alterations in mitochondrial respiratory complexes, we curated gene sets associated with this process and conducted a pre-ranked analysis using GSEA, as illustrated in Figure 3. Specifically, we focused on mitochondrial complex I activity using gene sets such as “MITOCHONDRIAL ELECTRON TRANSPORT NADH TO UBIQUINONE” from the Gene Ontology Biological Process (GOBP) database and “MITOCHONDRIAL COMPLEX I ASSEMBLY MODEL OXPHOS SYSTEM” from the WP database (Figure 3A). For complex III activity, we utilized gene sets “MITOCHONDRIAL COMPLEX III ASSEMBLY” from WP and “MITOCHONDRIAL ELECTRON TRANSPORT SUCCINATE TO UBIQUINONE” from GOBP. Regarding complex IV activity, we employed gene sets “MITOCHONDRIAL COMPLEX IV ASSEMBLY” from WP and “MITOCHONDRIAL ELECTRON TRANSPORT CYTOCHROME C TO OXYGEN” from GOBP. Notably, our analysis revealed a more pronounced suppression of mitochondrial complexes I in the aged skeletal muscle of rats, mice, and humans. This inhibition is associated with a decrease in NADH dehydrogenase activity, indicating dysregulation of the process leading to a decrease in ATP synthesis. Detailed representations of the enrichment regarding gene sets related to ATP synthesis and metabolic processes are presented in Figure 3B. Furthermore, gene sets associated with the mitochondrial translation process were consistently downregulated across species (Supplementary Figure S2). Overall, these findings collectively underscore the comprehensive dysregulation of mitochondrial functions in aged skeletal muscle.

### 2.4. Inflammatory Responses of IFN-γ, TNF-α, and IL-6 Were Central Factors That Induced Abnormal Inflammatory Responses across Species

**Figure 4:**
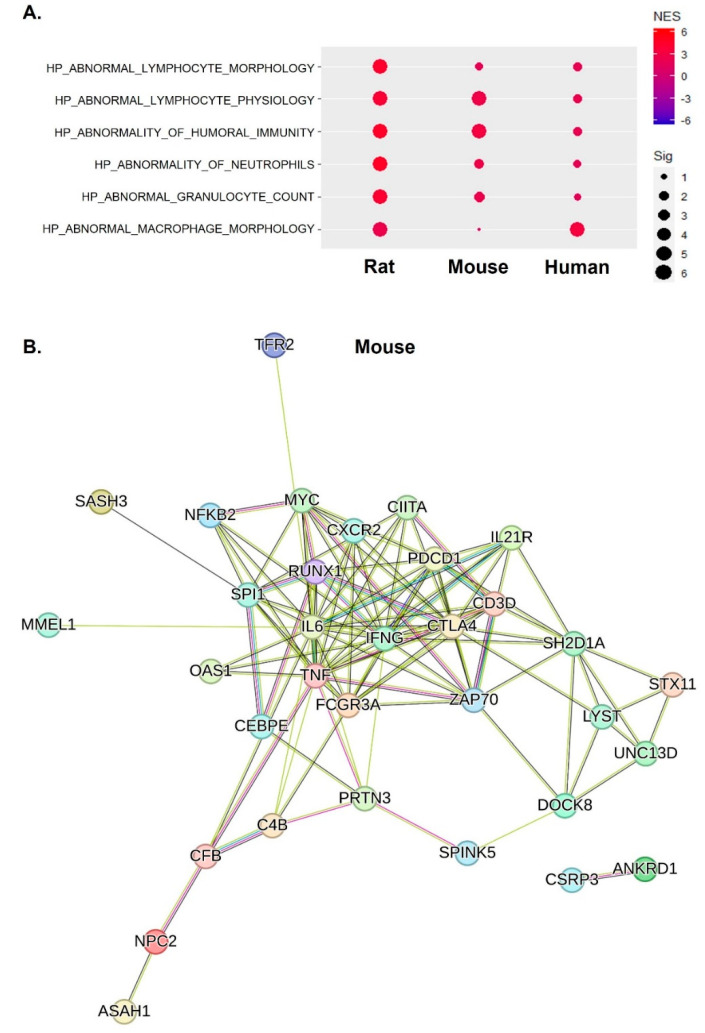

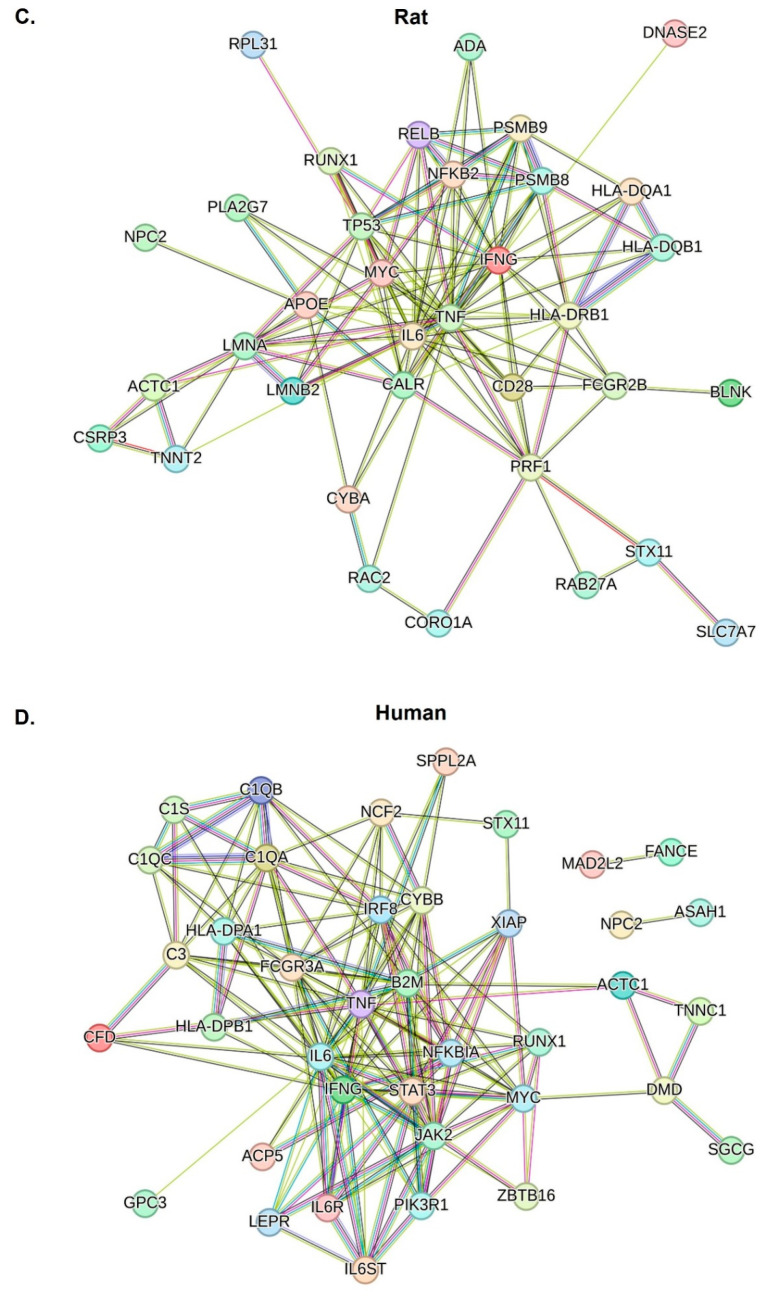
Abnormal inflammatory responses and the central role of IFN-γ, TNF-α, and IL-6 in sarcopenia across species. (**A**) Dot plots demonstrating the enrichment of gene sets related to abnormal inflammatory responses across species. (**B**–**D**) Network analysis showing the central role of IFN-γ, TNF-α, and IL-6 in abnormal inflammatory responses across species.

We examined the abnormality of inflammatory responses in aged skeletal muscles by leveraging MSigDB, and the significantly altered gene sets are depicted in Figure 4A. Across species, consistent positive enrichment patterns were observed, which were characterized by mechanisms such as abnormal lymphocyte morphology and physiology, humoral immunity, and innate immune responses involving macrophages, neutrophils, and granulocytes. A detailed GSEA graph explaining the enrichment is shown in Supplementary Figure S3, and although there are significant differences for each species, all of the inflammation-related gene sets mentioned above appear to be consistently increased by aging. In the network analysis of enriched gene sets related to abnormal inflammatory responses, IFN-γ, TNF-α, and IL-6 were centrally localized within the gene network, and this was found to be in common across all the studied species, as illustrated in Figure 4B–D. This central localization underscores the pivotal role of these key inflammatory mediators in the context of abnormal inflammatory responses in aged skeletal muscles.

### 2.5. Increased and Dysregulated Macrophage Function Was Commonly Detectable across Species

**Figure 5:**
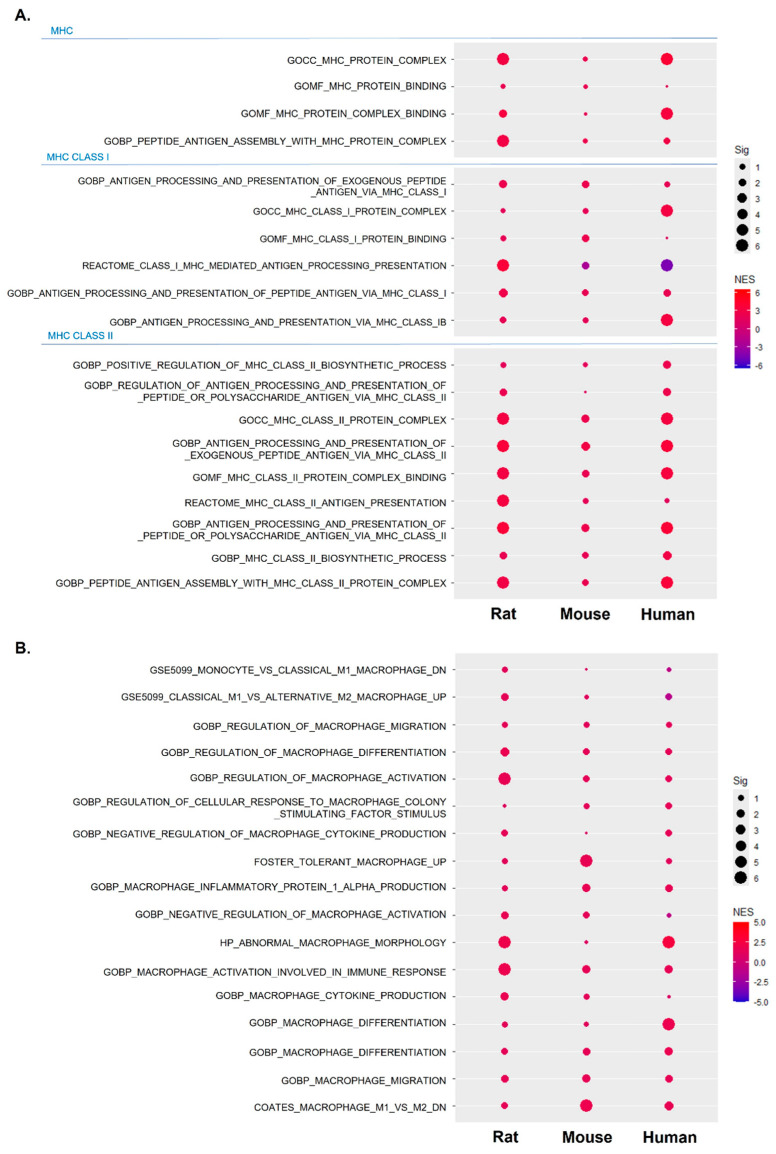
Activation and potential dysregulation of MHC class II expressing macrophages in sarcopenia across species. (**A**) Dot plots demonstrating the enrichment of gene sets related to MHC class I and II across species. (**B**) Dot plots demonstrating the enrichment of gene sets related to macrophage activation and abnormality across species.

Based on the results in Figure 4, we hypothesized that the dysregulated function of skeletal muscle-resident macrophages may serve as an important indicator of sarcopenia, and as shown in Figure 5, we systematically investigated macrophage function across different species. First, the MHC protein complex and binding were activated across species (Figure 5A). A notable observation in Figure 5A is the more pronounced activation of the MHC class II response compared to the MHC class I response, which aligns with previous findings that link chronic expression of IFN-γ to the activation of MHC class II response [16]. By extending the analysis and further analyzing macrophage-related gene sets, we found that gene sets related to macrophage activation, differentiation, migration, production, and dysregulation were activated (Figure 5B). Despite an overall positive enrichment pattern across species, the observed patterns were not as robust as those related to abnormality. This suggests a potential dysregulation in macrophage function, particularly in M1 macrophages, emphasizing the need for further investigation to improve our understanding of the nuanced aspects of macrophages’ involvement in sarcopenia.

## 3. Discussion and Conclusions

We recently reported that obesity-induced inflammation specifically dysregulated mitochondrial complex I activity in liver and adipose tissues, possibly through the chronic expression of IFN-γ according to the longitudinal study of high-fat diet-fed mice [17,18]. In line with previous findings, our study shows that the kinetics observed in obesity also apply to the development of sarcopenia. Considering the correlation between dysregulated mitochondrial complex I activity and macrophage dysfunction, which triggers heightened inflammatory responses in obesity conditions, our data support that these observations in the skeletal muscle are key pathogenic markers for sarcopenia.

The accumulation of dysfunctional mitochondria initiates the apoptotic death signaling pathway in muscle cells and motor neurons, and communication between these cell types accelerates muscle wasting and denervation and leads to sarcopenia [19]. Consistently, our hallmark gene set analysis using DEGs demonstrated that apoptosis in skeletal muscle was observed across species, along with features of dysfunctional mitochondria, supporting the theory that increased apoptosis is associated with the accumulation of mitochondrial abnormalities in sarcopenia to accelerate muscle wasting and denervation.

Considering the widely acknowledged role of mitochondrial DNA (mtDNA) mutations in sarcopenia [20], it is plausible that dysregulated mitochondrial gene expression contributes to a reduction in mitochondrial biogenesis and an increase in mtDNA mutations. Consequently, the disruption in mitochondrial function may catalyze the induction of mtDNA mutations within the mitochondrial respiratory chain reactions. This connection underscores the potential involvement of the mitochondrial respiratory chain in initiating mtDNA mutations when mitochondrial function is compromised due to the dysregulation of mitochondrial respiratory chains.

In the context of sarcopenia, chronic inflammation is often observed, and dysfunctional macrophages may contribute to this inflammatory environment. Persistent inflammation can negatively impact muscle health, potentially accelerating the degenerative processes associated with sarcopenia, such as muscle fiber loss and impaired regeneration [21]. Moreover, dysfunctional macrophages may fail to efficiently clear cellular debris and support proper muscle regeneration, further exacerbating the progression of sarcopenia [22]. We also recognize the importance of immune system aging in sarcopenia and its links to various comorbidities. Several candidate immune response biomarkers, including TLR signaling and MHC, have been identified, showing strong correlations with sarcopenia. These biomarkers provide insight into the complex relationship between immune dysregulation, chronic inflammation, and compromised tissue repair, highlighting potential mechanistic roles in the development or worsening of sarcopenia. Understanding these mechanisms is crucial for identifying potential therapeutic targets to mitigate the impact of immune-related factors on muscle health.

In our earlier study, we posited that chronic inflammation, specifically that initiated by IFN-γ, served as the primary pathogenic factor contributing to the suppression of mitochondrial complex I activity in the context of obesity [18]. Despite the essential role of complex I activity in eliciting IFN-γ responses, our longitudinal analysis revealed that sustained, albeit low, and chronic expression of IFN-γ emerged as a potential trigger for the abrupt dysfunction of complex I activity to suppress the IFN-γ response. The results of this study also suggest that chronic expression of IFN-γ may contribute to the suppression of complex I activity. Consequently, this downregulation of complex I activity may lead to a dampening of the IFN-γ response, particularly in M1 macrophages. This potential interplay suggests a dynamic relationship in which the chronic expression of IFN-γ may modulate complex I activity, influencing the preferential occurrence of the IFN-γ response in specific macrophage subsets during the development of sarcopenia.

During immune responses, cells may shift their metabolism to prioritize glycolysis over OXPHOS [23]. If OXPHOS is suppressed or disrupted, there may be a reduction in the flow of electrons through the ETC. A decrease in electron flow can lead to an accumulation of electrons in certain ETC complexes, potentially increasing the likelihood of electron leakage and ROS production. Our data reveal a notable suppression of mitochondrial respiratory complexes, particularly complex I and III, suggesting a pronounced vulnerability to excessive reactive oxygen species (ROS) production. This vulnerability can lead to a vicious cycle where increased levels of ROS contribute to damage to and dysfunction of mitochondrial complexes. As mitochondrial complex I components are encoded by mitochondrial DNA (mtDNA) [24], mitochondrial damage can lead to the leakage of mtDNA into the cytosol, further exacerbating complex I abnormalities.

While our results align with previous studies that demonstrated a decrease in M1 macrophages with aging [25,26], it is more likely that dysregulated macrophage function plays a more critical role in the pathogenesis of sarcopenia. Macrophages are crucial in regulating the inflammatory response during muscle damage. In sarcopenia, impaired macrophage function can lead to an excessive and prolonged inflammatory response. Instead of resolving inflammation and promoting tissue repair, dysfunctional macrophages may sustain a chronic inflammatory state, accelerating muscle degradation. This persistent inflammation can increase muscle protein breakdown and inhibit muscle regeneration, thereby exacerbating the loss of muscle mass and the strength characteristics of sarcopenia. The immunometabolic switch between M1 and M2 macrophages alone is insufficient to fully explain sarcopenia’s complex pathogenesis [27]; rather, the lack of appropriate immune responses to muscle damage may significantly contribute to compromised muscle health.

## 4. Methods

### 4.1. Gene Expression Data Acquisition

Utilizing the GEO database, we conducted a comparative analysis of gene expression profiles associated with age-related sarcopenia in humans (GSE136344), rats (GSE38077), and mice (GSE125815). In GSE136344, the transcriptomic data were obtained from the vastus lateralis muscle of young men and older men aged over 70 without metabolic syndrome, while GSE38077 investigated the gastrocnemius muscle of 24-month-old rats. GSE125815 focused on the gastrocnemius muscle of 26-month-old male mice. Differential expression analysis was carried out using the Gene Expression Omnibus 2R (GEO2R), an interactive web tool provided by the National Center for Biotechnology Information (NCBI). GEO2R employs the t-test as its primary statistical method for comparing means between two groups.

### 4.2. Metabolome Data Acquisition

To comprehensively analyze the metabolome, we integrated data from two referenced studies [28,29]. In one investigation, plasma metabolites were examined in a group of twenty elderly men (mean age: 81.9 ± 2.8 years). They were categorized into two groups: individuals with severe sarcopenia (*n* = 10) and non-sarcopenic controls (*n* = 10). Matching for age and body mass index was carried out according to the criteria outlined by the Asian Working Group for Sarcopenia 2019 [28]. The other study involved metabolomic profiling of the skeletal muscle in both young (8-week-old, *n* = 5) and aged (28-month-old, *n* = 5) C57BL/6J mice [29]. The significant metabolites identified in these studies were further analyzed in conjunction with inflammatory mediators such as IL-10, TLR4, IL-6, TNF, and IFN-γ. Protein–protein interactions were elucidated using STRING (https://string-db.org, accessed on 20 August 2024), and the resulting network was further visualized using Cytoscape version 3.10.1.

### 4.3. Pathway Analysis

For user-friendly pathway analysis based on differentially expressed genes (DEGs), we utilized Enrichr and g: profiler. The first step in our pathway analysis encompassed the meticulous curation of a DEG list, adhering to stringent criteria such as a log-fold change cutoff of 2 and a significance level less than 0.05. The DEGs were identified using the GEO2R statistical tool, which allows for the comparison of two or more groups of samples within the GEO database. Next, we conducted a Pre-ranked Gene Set Enrichment Analysis (GSEA), leveraging whole gene expression data. This approach involved ranking gene expression profiles based on fold changes, allowing us to assess gene set enrichment comprehensively. The GSEA algorithm is subsequently applied to evaluate gene set enrichment, based on normalized enrichment scores (NES) and enrichment scores (ES). A positive NES and ES indicate that a gene set is positively enriched, meaning it is upregulated in the condition of interest. Conversely, a negative NES and ES suggest that the gene set is negatively enriched, reflecting downregulation in the condition. Significance is typically assessed using various criteria: the nominal p-adjusted value, False-Discovery Rate (FDR), and Family-Wise Error Rate (FWER), which were commonly set below 0.05. In our analysis, we chose to emphasize the significance assessment based on FDR. However, for individual analyses, we considered all criteria, since FDR may not be applicable.

### 4.4. Data Visualization

Our data visualization endeavors were primarily executed using R version 4.3.1 and RStudio version 2022.12.0 + 353, which served as our principal software tools. Additionally, we integrated Appyters, a web-based platform designed to streamline user-friendly and efficient visualization processes (CC-BY-NC-SA-4.0). This combination of versatile R packages and the accessibility offered by Appyters ensured a comprehensive and accessible approach to creating informative and visually appealing graphics.

## Data Availability

The datasets generated during and/or analyzed during the current study are not publicly available but are available from the corresponding author on reasonable request.

## Supplementary Materials

The following supporting information can be downloaded at https://www.mdpi.com/article/10.3390/ijms251910302/s1.
