# Supplementary material for: Cross-Species Studies Reveal That Dysregulated Mitochondrial Gene Expression and Electron Transport Complex I Activity Are Crucial for Sarcopenia
Source: Int J Mol Sci. 2024 Sep 25;25(19):10302. doi: 10.3390/ijms251910302 (PMC11477305; doi:10.3390/ijms251910302)
Supplement: Supplementary file 1 [file ijms-25-10302-s001.zip › ijms-3191055-supplementary.pdf]

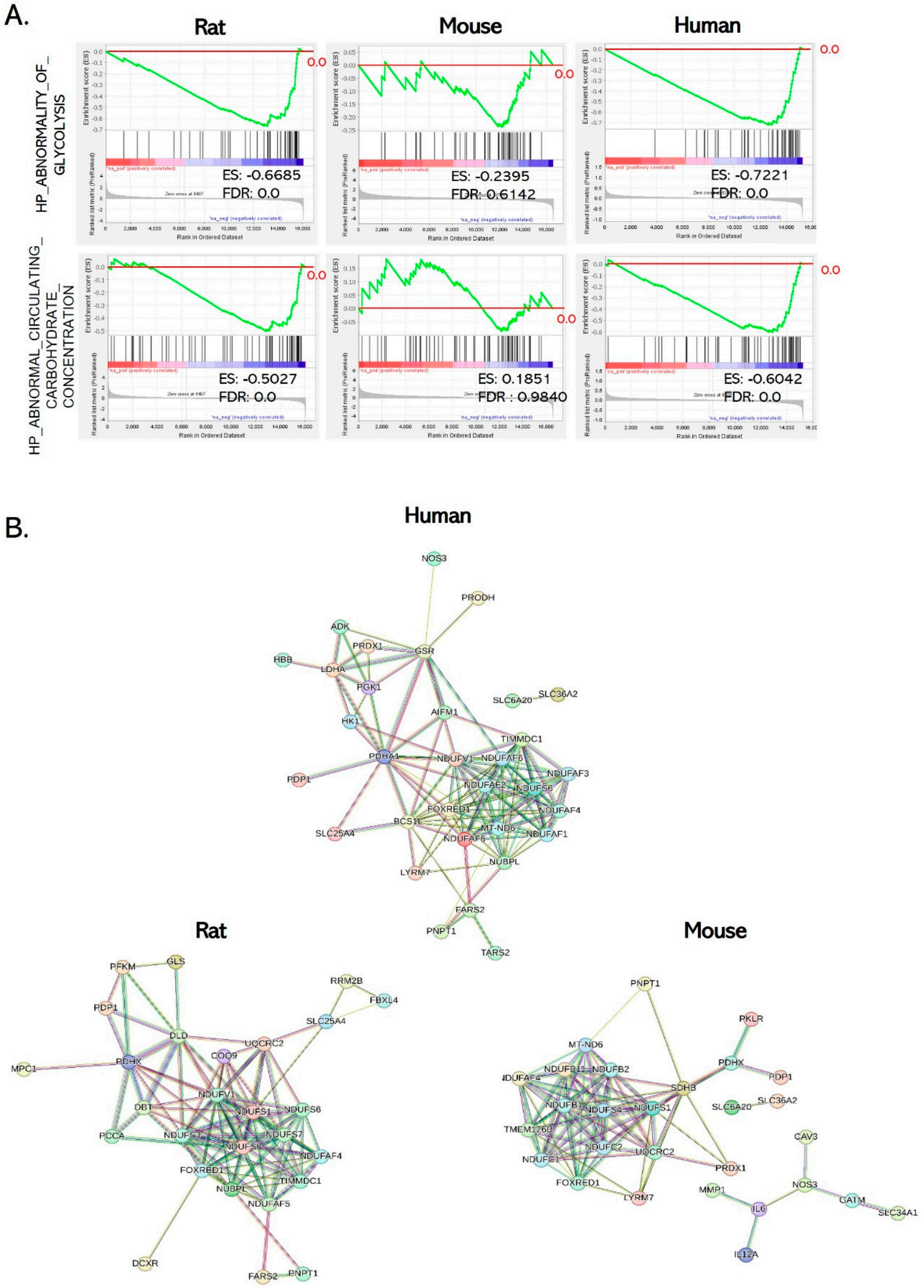

Supplementary Figure S1. Gene interaction between abnormal metabolic processes and mitochondrial complex I activity in sarcopenia across species. (a) Representative plots showing enrichment in abnormal metabolic processes generated from GSEA across species. (b) Network analysis showing gene interaction between mitochondrial respiratory complex I and abnormal metabolic processes. The yellow dot indicates genes involved in mitochondrial complex I activity.

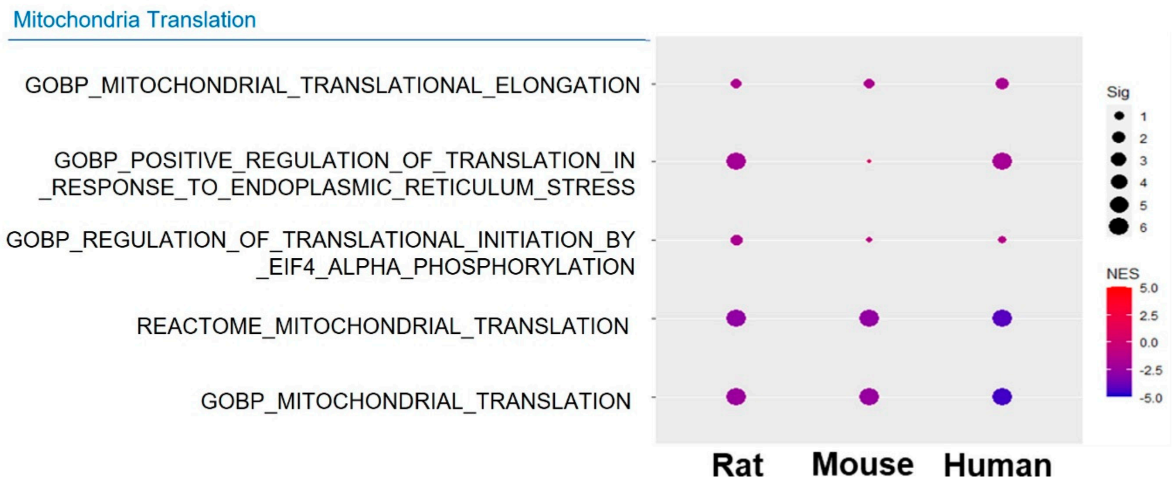

Supplementary Figure S2. Suppressed mitochondrial translation in sarcopenia across species. Representative plots showing enrichment of gene sets related to mitochondrial translation processes generated from GSEA in sarcopenia across species.

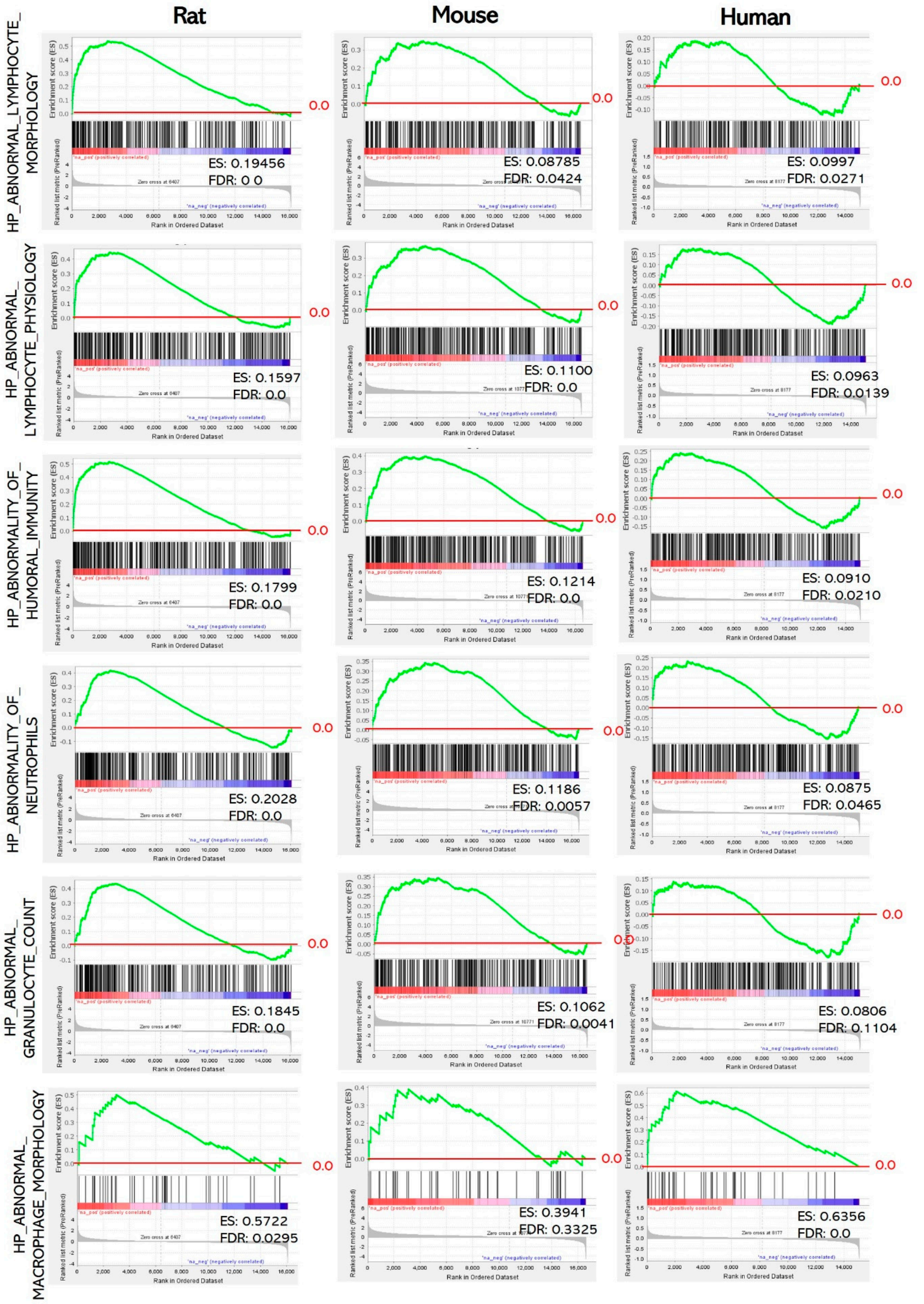

Supplementary Figure S3. Abnormal inflammatory responses in sarcopenia across species. Representative graphs showing enrichment of gene sets associated with abnormal inflammatory responses, generated from GSEA, in sarcopenia across species.
